# Structural Biology of DNA (6-4) Photoproducts Formed by Ultraviolet Radiation and Interactions with Their Binding Proteins

**DOI:** 10.3390/ijms151120321

**Published:** 2014-11-06

**Authors:** Hideshi Yokoyama, Ryuta Mizutani

**Affiliations:** 1 School of Pharmaceutical Sciences, University of Shizuoka, 52-1 Yada, Suruga-ku, Shizuoka 422-8526, Japan; 2 Department of Applied Biochemistry, School of Engineering, Tokai University, Kanagawa 259-1292, Japan; E-Mail: ryuta@tokai-u.jp

**Keywords:** DNA damage, DNA (6-4) photoproduct, ultraviolet light, nucleotide flipping, crystal structure, antigen-binding fragment, antibody, photolyase, nucleotide excision repair

## Abstract

Exposure to the ultraviolet component of sunlight causes DNA damage, which subsequently leads to mutations, cellular transformation, and cell death. DNA photoproducts with (6-4) pyrimidine-pyrimidone adducts are more mutagenic than cyclobutane pyrimidine dimers. These lesions must be repaired because of the high mutagenic potential of (6-4) photoproducts. We here reviewed the structures of (6-4) photoproducts, particularly the detailed structures of the (6-4) lesion and (6-4) lesion-containing double-stranded DNA. We also focused on interactions with their binding proteins such as antibody Fabs, (6-4) photolyase, and nucleotide excision repair protein. The (6-4) photoproducts that bound to these proteins had common structural features: The 5'-side thymine and 3'-side pyrimidone bases of the T(6-4)T segment were in half-chair and planar conformations, respectively, and both bases were positioned nearly perpendicularly to each other. Interactions with binding proteins showed that the DNA helices flanking the T(6-4)T segment were largely kinked, and the flipped-out T(6-4)T segment was recognized by these proteins. These proteins had distinctive binding-site structures that were appropriate for their functions.

## 1. Introduction

**Figure 1:**
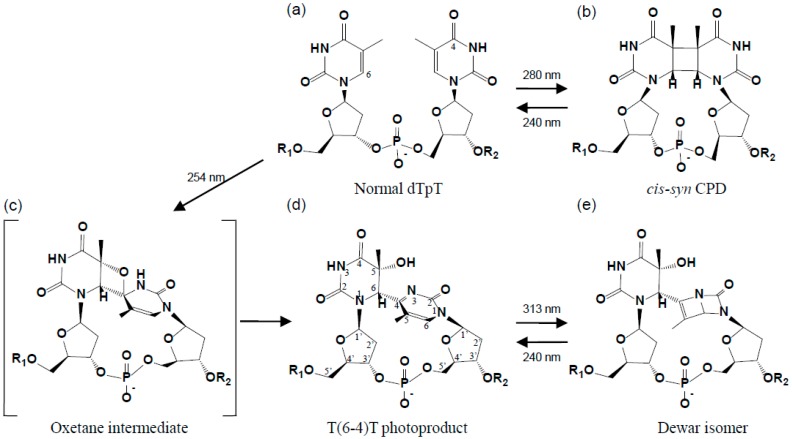
DNA photoproducts formed by ultraviolet radiation. (**a**) Normal dTpT; (**b**) *cis-syn* cyclobutane pyrimidine dimer (CPD); (**c**) Oxetane intermediate; (**d**) A dT(6-4)T photoproduct with atom numbering; and (**e**) Dewar isomer.

Exposure to the ultraviolet component of sunlight causes DNA damage, which subsequently leads to mutations, cellular transformation, and cell death [1]. The photo-damaged DNAs produced from two adjacent pyrimidine bases include *cis-syn* cyclobutane pyrimidine dimers (CPDs), pyrimidine-pyrimidone (6-4) photoproducts, and Dewar isomers (Figure 1). CPD and (6-4) photoproduct comprise 70%–80% and 20%–30%, respectively, of the total photoproducts [2]. T(6-4)T photoproducts frequently cause T-to-C mutations at their 3'-sides during the replication of DNA, and thus are more mutagenic [3]. Deficiencies in the repair of DNA photoproducts in humans lead to a hereditary disease called xeroderma pigmentosum [4]. Xeroderma pigmentosum patients are extremely sensitive to sunlight and are at an approximately 10^3^-fold higher risk of developing skin cancer [5]. The toxic effects of UV-induced DNA damage were found to be reduced *in vivo* following the repair of photolesions using DNA photolyases and nucleotide excision repair enzymes [6]. The structures of CPDs and reaction mechanisms underlying their repair enzymes have been investigated extensively [7]. However, structural studies on (6-4) photoproducts and their binding proteins have been conducted less frequently than those on CPDs. In this review, we focus on the structures of (6-4) photoproducts and their binding proteins.

In order to detect and quantify the (6-4) photoproduct, a series of monoclonal antibodies, 64M-2, 64M-3, and 64M-5, have been simultaneously established from BALB/c mice immunized with ultraviolet-irradiated, single-stranded calf thymus DNA [8]. These antibodies are highly specific to the (6-4) photoproduct and show no affinity to other damaged or undamaged DNA [9]. The findings of a kinetic study of the 64M-5 Fab using single-stranded (6-4) photoproducts of various lengths indicated higher affinity for longer oligonucleotides, up to the hexamer, with each containing the dT(6-4)T segment in the middle [9]. An NMR study [10] reported that four phosphate groups on both sides of the dT(6-4)T segment were involved in the interaction with the 64M-5 Fab. In order to elucidate the structural recognition of (6-4) photoproducts by the antibody, we determined the crystal structure of dT(6-4)T or dTT(6-4)TT as a complex with the Fab fragment of the 64M-2 antibody, which was highly homologous to the 64M-5 antibody [11,12]. The antibody 64M-5, which exhibited the highest affinity toward the (6-4) photoproduct among the 64M-2, 64M-3, and 64M-5 antibodies, was originally produced against single-stranded (6-4) DNA [8], and was later found to bind double-stranded (6-4) DNA [13]. We determined the crystal structure of the 64M-5 Fab in complex with double-stranded DNA containing a T(6-4)T segment in order to elucidate the structural basis for why 64M-5 could also bind to double-stranded (6-4) DNA [14].

The (6-4) photoproduct must be repaired because of its high mutagenic potential. This photoproduct can be repaired by photoreactivation and nucleotide excision repair. (6-4) Photolyase, a photoreactivating enzyme, recognizes the (6-4) photoproduct and has been shown to restore damaged bases to their native form in a light-dependent reaction [6,15]. (6-4) Photolyases repair the (6-4) photoproduct with a small quantum yield (approximately 0.1), while CPD photolyases repair CPD with a high quantum yield (approximately 0.7) [16,17,18,19]. Several groups have proposed the reaction mechanisms of (6-4) photolyases [20,21,22,23,24]. In nucleotide excision repair of the eukaryotic type, DNA lesions including (6-4) photoproducts are removed by hydrolyzing the 20th–25th phosphodiester bond 5' and the 3rd–8th phosphodiester bond 3' to the lesion [25]. Damage and structural distortion are recognized and excised as a fragment of DNA, and the resulting gap is filled in by a DNA polymerase [26]. The crystal structures of the DNA (6-4) photolyase [27,28] and nucleotide excision repair protein DDB1-DDB2 [29] have been determined as a complex with a DNA duplex containing a central (6-4) lesion. These structures suggest that recognition of the (6-4) lesion is achieved by a structural change in (6-4) lesion-containing DNA. We also discuss the recognition mechanism of the T(6-4)T segment by these repair enzymes.

## 2. Structure of the (6-4) Photoproduct

### 2.1. Structure of dT(6-4)T

**Figure 2:**
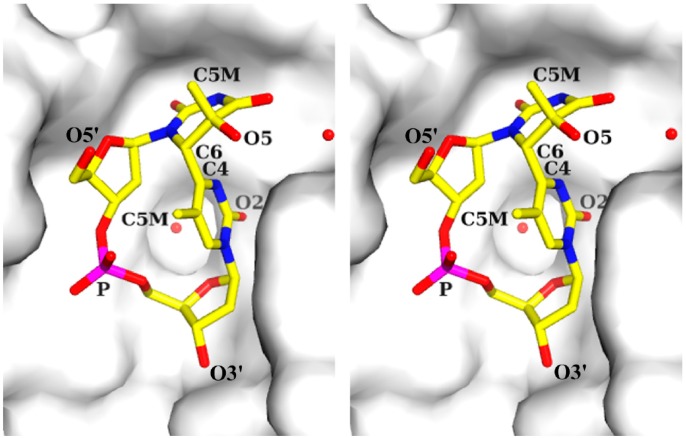
Stereo drawing of the structure of dT(6-4)T in the crystal structure of the 64M-2 Fab—dT(6-4)T complex (PDB ID: 1EHL). The dT(6-4)T molecule is shown as a stick model (C atoms, yellow; N atoms, blue; O atoms, red; P atoms, magenta), and several atoms were numbered. The Fab is represented as a grey surface model. Water molecules are drawn with red spheres. All molecular figures in this study were produced with PyMOL [35].

The (6-4) photoproduct is characterized by the formation of a covalent bond between two adjacent pyrimidine bases: C6 of the 5'-base and C4 of the 3'-base (Figure 1d). The photochemical generation of the (6-4) photoproduct involves the transfer of the hydroxyl group at C4 of the 3'-base, via an oxetane intermediate (Figure 1c), to the C5 position of the 5'-base [6]. The thymine-pyrimidone base moiety of dT(6-4)T has a chemical structure of 5-hydroxy-6-4'-(5'-methylpyrimid-2'-one)-dihydrothymine [30], and the crystal structure of its base moiety was published in 1969 [31,32]. The crystal structure of (6-4) photoproducts was not subsequently examined for approximately 30 years after this structure was published. Our group published the crystal structure of dT(6-4)T in 2000 [11], and showed that it was the whole segment of a dinucleotide and consisted of a base moiety, 5'-deoxyribose, 3'-deoxyribose, and the connecting phosphate group (Figure 2). The crystal structure of dT(6-4)T was determined in complex with the antibody Fab fragment. The dT(6-4)T is accommodated in the antigen-binding pocket of the Fab. The solution structures of dT(6-4)T, a dinucleotide containing a T(6-4)T segment, have also been determined in a protein-free isolated form [33,34].

In the crystal structure of the 64M-2 Fab—dT(6-4)T complex [11], dT(6-4)T adopted a closed circular structure by forming a covalent bond between the C6 atom of the 5'-base and C4 atom of the 3'-base (Figure 2), and the (6-4) linkage restricted the conformational flexibility of dT(6-4)T. Hence, the rigidity of the dT(6-4)T molecule appeared to be higher than that of a normal thymidine dimer. The thymine-pyrimidone base moiety of dT(6-4)T in the 64M-2 Fab—dT(6-4)T complex [11] adopted almost the same structure as the crystal structure of the base moiety [31]. The root-mean-square difference (r.m.s.d.) between the protein-bound dinucleotide and free base-moiety structures was 0.16 Å. This result indicated the intrinsic rigidity of the (6-4) covalent linkage. The N1−C6−C4#−C5# torsion angle of the (6-4) covalent linkage was −139° (Table 1: The symbol # means the 3'-pyrimidone base; the torsion angle defined as in [36].) The angle between the 5'-thymine plane, defined as the mean plane for the six-membered 5'-thymine atoms, and the 3'-pyrimidone plane, which was similarly defined, was 99°, indicating that these planes were nearly perpendicular to each other. The angle between the 5'-thymine and 3'-pyrimidone planes was 96° in the crystal structure of the base moiety [31]. The 5'-thymine base was in the half-chair conformation in which the C5 atom was placed above the mean plane and the C6 atom below (Figure 2). Both the C5 methyl group and 3'-pyrimidone ring were axial to the 5'-thymine ring, and placed opposite to each other, with a (C5 methyl)-C5-C6-C4# torsion angle of −164° (Table 1). The O5 atom on C5 of the 5'-thymine was equatorial. The 3'-pyrimidone base was nearly planar and its C5 methyl group pointed toward the 5'-deoxyribose ring. Both the 5'-thymine N1 and 3'-pyrimidone N1 atoms showed planar *sp*^2^ configurations.

**Table 1:** DNA torsion angles.

Segment	Definition	Torsion Angle (°)		
		64M-2 Fab dT(6-4)T ^a^	dT(6-4)T ^a^	B-DNA ^b^
(6-4) Linkage ^c^	N1−C6−C4#−C5#	−139	−	
	N1−C6−C4#−N3#	39	27	
	(C5 methyl)−C5−C6−C4#	−164	−163	
**5'-T**				
Glycosidic linkage	χ (O4'−C1'−N1−C2)	−135	−138	−117
Backbone	γ (O5'−C5'−C4'−C3')	103	56	54
	δ (C5'−C4'−C3'−O3')	85	80	123
	ε (C4'−C3'−O3'−P)	−110	−107	−169
	ζ (C3'−O3'−P−O5')	−84	−76	−108
**3'-T**				
Glycosidic linkage	χ (O4'−C1'−N1−C2)	−71	−75	−117
Backbone	α (O3'−P−O5'−C5')	−71	−96	−63
	β (P−O5−C5'−C4')	−172	−177	171
	γ (O5'−C5'−C4'−C3')	35	43	54
	δ (C5'−C4'−C3'−O3')	120	100	123

^a^ The torsion angles of dT(6-4)T are shown for the 64M-2 Fab—dT(6-4)T complex [11] and isolated dT(6-4)T in solution [34]; ^b^ The mean torsion angles of double-stranded B-DNA, the so-called Drew-Dickerson dodecamer [37], are also shown as a reference; and ^c^ The atoms of 3'-T are marked with #.

**Figure 3:**
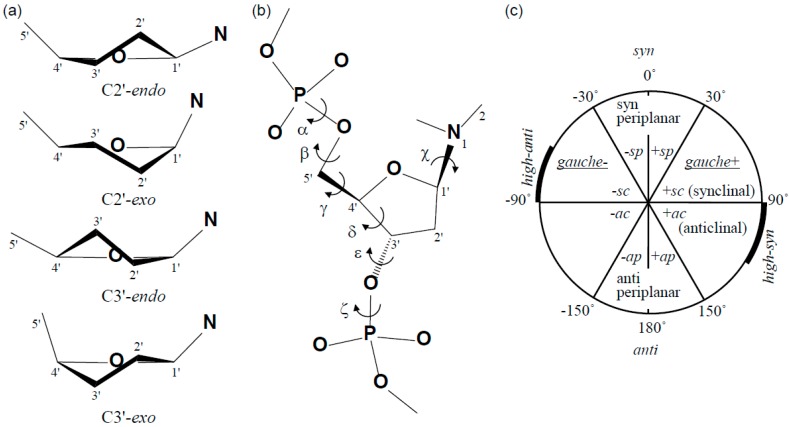
Schematic representation of DNA sugar puckers (**a**) and torsion angles (**b**); and the range of torsion angles (**c**). These figures were produced based on a previous study [36].

In the 64M-2 Fab—dT(6-4)T complex [11], 5'-deoxyribose was in the C3'-*endo* conformation and 3'-deoxyribose was nearly planar (Figure 2 and Figure 3a). An NMR study on the conformation of dT(6-4)T [33] suggested a two-state model in which the 3'-deoxyribose ring equilibrated between C3'-*endo* (60% occupancy) and C3'-*exo* (40%) puckers. Another NMR study [34] indicated that the 3'-deoxyribose ring was either in equilibrium between C2'-*exo* (70%) and C3'-*exo* (30%) puckers or in the single state only of a C1'-*endo* pucker. Therefore, the flexibility of 3'-deoxyribose could be observed in dT(6-4)T isolated in solution. The 3'-deoxyribose of bound dT(6-4)T was tightly packed to the Fab surface and was in a nearly planar conformation; however, this conformation was generally unfavorable for the deoxyribose [36]. The planar 3'-deoxyribose of dT(6-4)T bound to the 64M-2 Fab was fixed in the intermediate between the C3'-*endo* and C3'-*exo* conformations. The 5'-deoxyribose of the bound dT(6-4)T was in the C3'-*endo* conformation, while the deoxyribose of B-DNA was in the C2'-*endo* conformation [37]. The two NMR studies on the conformation of dT(6-4)T [33,34] also suggested that the 5'-deoxyribose ring may have the C3'-*endo* conformation. The conversion of C2'-*endo* to C3'-*endo* brought the adjacent bases closer [36]. Therefore, the C3'-*endo* conformation was attributed to the formation of the (6-4) linkage.

The backbone torsion angles α, β, γ, δ, ε, and ζ of 5'-deoxyribose, phosphate, and 3'-deoxyribose (Table 1 and Figure 3b) indicated a backbone conformation similar to that of the standard B-DNA structure [37,38]. The largest difference between the dT(6-4)T and B-DNA structures was observed in the torsion angle ε (C4'-C3'-O3'-P) of 5'-deoxyribose. The ε values of dT(6-4)T in the 64M-2 Fab—dT(6-4)T and dT(6-4)T isolated in solution were approximately 60° larger than that of the B-DNA. This difference corresponded to the rotation angle that superposed the 3'-pyrimidone ring onto the 5'-thymine ring. The torsion angles of dT(6-4)T in the 64 M-2 Fab—dT(6-4)T complex were almost the same as those in dT(6-4)T isolated in solution, except for the torsion angle γ (O5'−C5'−C4'−C3') of 5'-thymine (Table 1). The torsion angle γ around the C4'-C5' bond of deoxyriboses characterized the orientation of O5' relative to the deoxyribose ring (Figure 3b). The terminal O5' atom of 5'-thymine may have been relatively flexible, and, thus, the torsion angle γ may have slightly differed between 64M-2 Fab—dT(6-4)T and isolated dT(6-4)T. The torsion angle χ (O4'−C1'−N1−C2) was the angle around the glycosyl bond to the deoxyribose ring (Figure 3b). 5'-Thymine with a glycosidic torsion angle χ of −135° was in an *anti* conformation, while 3'-pyrimidone with an angle of −71° was in a *high-anti* (-*sc*) conformation [36] (Table 1 and Figure 3c). These values were close to those (−138° and −75°) for dT(6-4)T isolated in solution (Table 1). These χ angles differ from those of B-DNA (Table 1), and the χ angles of the 3'-pyrimidone, in particular, were approximately 40° larger than those of B-DNA. If the χ angles of 3'-pyrimidone were the same as those of the B-DNA, the C5 methyl group of 3'-pyrimidone may have caused steric hindrance to 5'-deoxyribose. These typical χ angles were attributed to the formation of the (6-4) linkage that connects 5'-thymine perpendicularly to 3'-pyrimidone. The phosphodiester torsion angles ζ (C3'–O3'–P–O5') and α (O3'–P–O5'–C5') in the 64M-2 Fab—dT(6-4)T complex were in the -*sc* (*gauche*-) range. These angles were close to those in dT(6-4)T isolated in solution.

### 2.2. Structure of Double-Stranded DNA Containing T(6-4)T

**Figure 4:**
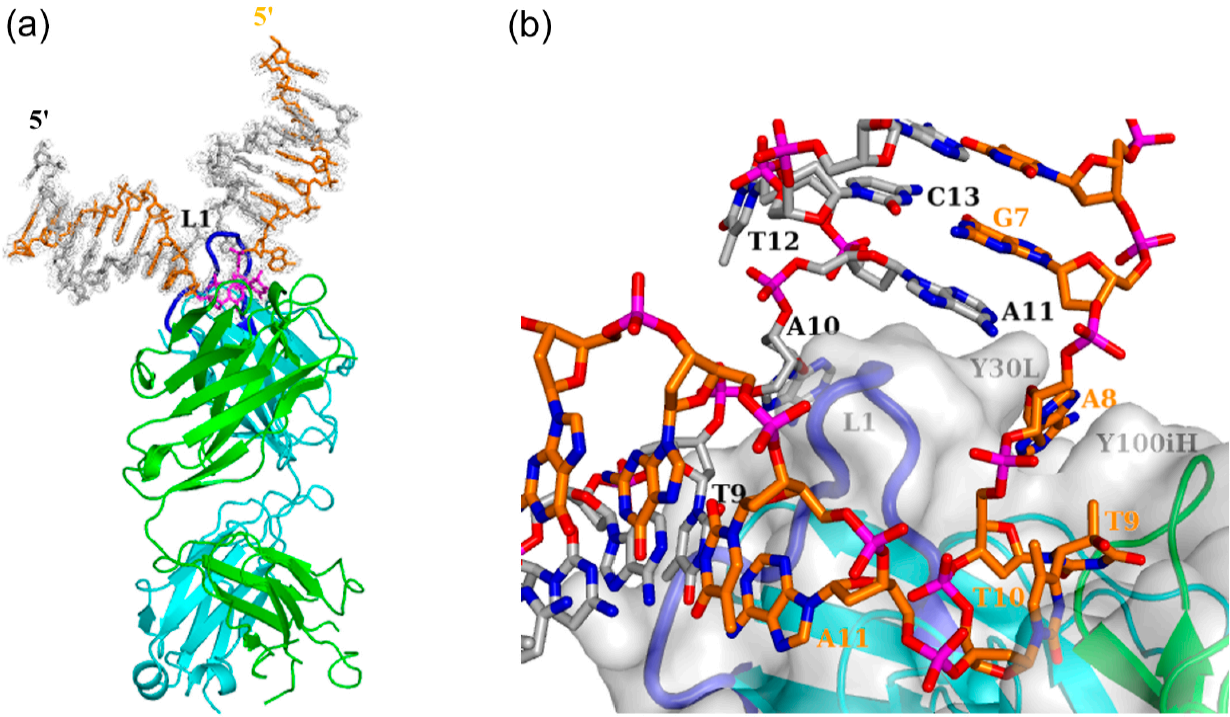
Overall structure of the 64M-5 Fab—ds-(6-4) DNA complex (PDB ID: 3VW3). (**a**) DNA is shown as stick models with the omit *F*_o_–*F*_c_ electron density map (1.5 σ level). The T(6-4)T segment is drawn in magenta, the T(6-4)T-containing strand in orange (the 5'-end is labeled in orange), and the complementary strand in grey (the 5'-end is labeled in black). The heavy chain of Fab is drawn in green, and the light chain in cyan. The CDR L1 loop inserted between the T(6-4)T-containing strand and the complementary strand is drawn in blue; and (**b**) Close-up view. The Fab is also represented as a grey surface model. The T(6-4)T-containing strand is labeled in orange, and the complementary strand in black. (Reproduced and slightly modified from reference [14] with the permission of the International Union of Crystallography.)

We recently described the crystal structure of double-stranded (ds) DNA containing a T(6-4)T segment in complex with the 64M-5 Fab (Figure 4) [14]. The central T(6-4)T segment of the 64M-5 Fab—ds-(6-4) DNA complex gave a small r.m.s.d. of 0.34 Å from that of the 64M-2 Fab—dT(6-4)T complex [11], and, thus, showed a similar structure. The central T(6-4)T segment of the 64M-2 Fab—dTT(6-4)TT complex [12] also gave a small r.m.s.d. of 0.31 Å from that of the 64M-2 Fab—dT(6-4)T complex. The six-membered ring planes of the central T(6-4)T bases were nearly perpendicular to each other, with an interplanar angle of 80° or 77° in the structures of the 64M-5 Fab—ds-(6-4) DNA or 64M-2 Fab—dTT(6-4)TT complex, respectively. In these two structures, the 5'-thymine bases of the central T(6-4)T segment were in the half-chair conformation, and the 3'-pyrimidone bases were in the planar conformation. These structural features were common to the 64M-2 Fab—dT(6-4)T complex, as described above. These results indicated that the dT(6-4)T structure was not affected by the flanking nucleotides.

The solution structure of the DNA duplex-decamer of d(CGCAT(6-4)TACGC)/d(GCGTAATGCG), which contained dT(6-4)T/dAA nucleotide pairs (underlined) in its center, was determined in the protein-free isolated form [39,40]. In this structure, the 3'-side pyrimidone base of the dT(6-4)T segment did not form a hydrogen bond with the complementary adenine base, while the 5'-side pyrimidine base of dT(6-4)T and the remaining nucleotides retained Watson-Crick-type hydrogen bonds with the complementary bases. Another solution structure of the DNA duplex-decamer of d(CGCAT(6-4)TACGC)/d(GCGTGATGCG), which contained dT(6-4)T/dGA unmatched nucleotide pairs in its center, was also determined [41]. In this structure, the 5'- and 3'-side T(6-4)T bases were hydrogen-bonded to the adenine and guanine bases, respectively, and the remaining nucleotides formed hydrogen bonds with the complementary bases: The carbonyl O2 of the 3'-side pyrimidone of T(6-4)T formed hydrogen bonds with the N1 and N2 protons of the opposed guanine base. The dT(6-4)T/dGA-containing duplex hence appeared to form more stable base-pairs than the dT(6-4)T/dAA-containing duplex, and provided an insight into understanding how T(6-4)T photoproducts frequently cause T-to-C mutations at their 3'-sides during the replication of DNA [3]. In these solution structures of the DNA duplex-decamer containing dT(6-4)T/dAA or dT(6-4)T/dGA, DNA duplexes flanking the T(6-4)T segment were kinked by 44° or 27°, respectively. On the other hand, the DNA duplex-decamer of d(CGCAT(*cis-syn*)TACGC)/d(GCGTAATGCG), which contained the *cis-syn* cyclobutane pyrimidine dimer and complementary AA nucleotides (underlined), was only kinked by 9° [40]. Both the 5'- and 3'-side T(*cis-syn*)T bases were hydrogen-bonded to two corresponding adenine bases, and the remaining nucleotides formed hydrogen bonds with the complementary bases. The DNA duplex showed no remarkable bending angle, compared to the T(6-4)T-containing DNA duplex-decamer. In the dT(6-4)T/dAA-containing duplex-decamer [39], the 3'-deoxyribose of the dT(6-4)T portion was restricted to the C2'-*exo* conformation. The flexibility of 3'-deoxyribose observed in isolated dT(6-4)T [33,34], as described above, was not allowed in the DNA duplex-decamer because of the steric hindrance caused by the adjacent nucleotides and complementary DNA.

In the crystal structures of double-stranded DNA containing the central T(6-4)T segment in complex with the DNA (6-4) photolyase [27] and nucleotide excision repair protein DDB1-DDB2 [29], two nucleotides of T(6-4)T were flipped-out of the duplex, and all remaining nucleotides retained Watson-Crick base pairing throughout the duplex. The DNA bending angles were 48° for (6-4) photolyase and 34° for DDB1-DDB2. These angles were similar to those of the solution structures of the DNA duplex-decamer containing T(6-4)T. Figure 4 shows the crystal structure of the 64M-5 Fab—ds-(6-4) DNA complex [14]. In this structure, 17 bp DNA with a one-base overhang of d(GCGAGTGAT(6-4)TATGGACGG)/d(CCCGTCCATAATCACTCG) was used. Three nucleotides of a 5'-side adenosine and T(6-4)T (A8, T9, and T10, labeled in red in Figure 4b) were flipped-out of the duplex, and all remaining nucleotide pairs formed standard B-DNA duplexes. The DNA helices flanking the T(6-4)T segment were kinked by 87°. The intensive interaction with the complementarity-determining region (CDR) L1 loop of the 64M-5 antibody should have accounted for the steep kink and resultant strand separation. The interactions between the DNA duplexes and these proteins have been described later.

## 3. Recognition Mechanism of the (6-4) Photoproduct by Proteins

### 3.1. Interaction of Antibody Fab with the (6-4) Photoproduct

#### 3.1.1. Fab Interaction with the T(6-4)T Segment

**Figure 5:**
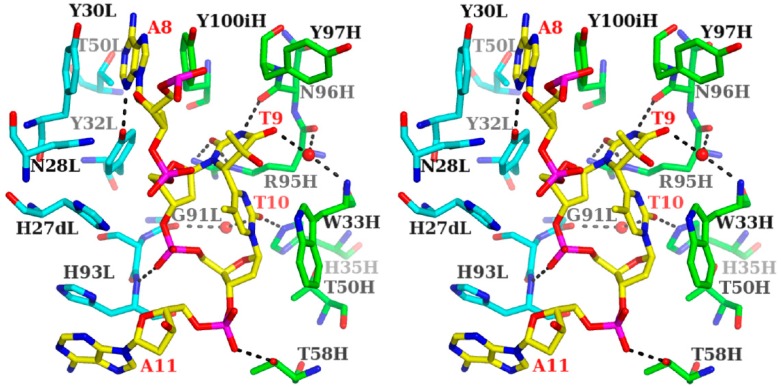
Stereo drawing of the antigen-binding site of the 64M-5 Fab—ds-(6-4) DNA complex (PDB ID: 3VW3). The AT(6-4)TA nucleotide of ds-(6-4) DNA is shown in yellow and labeled in red. The heavy chain of Fab is drawn in green, and the light chain in cyan. Hydrogen bonds are shown in broken lines. T9 and T10 correspond to the T(6-4)T segment. Water molecules are drawn with spheres.

In the crystal structures of the 64M-5 Fab—ds-(6-4) DNA complex [14] as well as the 64M-2 Fab—dT(6-4)T [11] and 64M-2 Fab—dTT(6-4)TT complexes [12], the T(6-4)T segment was accommodated in the antigen-binding pocket, as shown in Figure 4b. This pocket was approximately 15 Å long, 10 Å wide, and 8 Å deep, and was mainly constructed by basic and aromatic residues (Figure 5). The basic residues of His35H and Arg95H were located at the bottom of the pocket (light- and heavy-chain residues are numbered in accordance with Kabat *et al.* [42], and denoted by L and H suffixes, respectively). The aromatic residues of His27dL, Tyr30L, Tyr32L, His93L, Trp33H, Tyr97H, and Tyr100iH formed the side wall of the pocket. All six CDRs were involved in DNA recognition.

The T(6-4)T bases formed six hydrogen bonds with the Fab and water molecules. Regarding the 5'-thymine (T9 in Figure 5), the O2 atom was hydrogen-bonded to the Arg95H Nε and Nƞ2 atoms, and the N3 atom to Asn96H O. The O4 atom was hydrogen-bonded to a water molecule, which was hydrogen-bonded to the Trp33H N and Arg95H O atoms. As for 3'-pyrimidone (T10 in Figure 5), the O2 atom was hydrogen-bonded to His35H Nε2 and a water molecule, which was hydrogen-bonded to Gly91L O. Interactions between proteins and DNA are often mediated by ordered water molecules forming hydrogen-bonding networks. In the *trp* repressor-DNA complex [43], few direct contacts were detected between the protein and base pairs of DNA, while three ordered water molecules were located between the base pairs and protein side-chains. The two water molecules forming hydrogen-bonding networks in the 64M-5 Fab—ds-(6-4) DNA complex were also found in the structures of the 64M-2 Fab—dT(6-4)T (Figure 2) [11] and 64M-2 Fab—dTT(6-4)TT complexes [12], and, thus, commonly contributed to the specific recognition of the T(6-4)T segment. The side chains of Trp33H, Tyr97H, and Tyr100iH showed van der Waals interactions with the T(6-4)T bases. The 3'-pyrimidone base was in a stacking interaction with the indole ring of Trp 33H, with a face-to-face interplanar distance of 3.5 Å. The 64M-5 antibody single chain Fv mutants in which Trp33H was substituted with phenylalanine, tyrosine, or alanine, diminished antigen binding [44]. This finding indicated that Trp33H was involved in the specific recognition of the T(6-4)T segment. Hydrogen bonds by His35H and Arg95H and the stacking interaction by Trp33H have commonly been observed in the structures of the 64M-2 Fab—dT(6-4)T [11] and 64M-2 Fab—dTT(6-4)TT complexes [12]. The basic side-chains of His27dL and His93L were located at the side wall, and were in an electrostatic interaction with the phosphate group of the T(6-4)T segment. Furthermore, the O1P atom of the phosphate group was hydrogen-bonded to His93L N.

The 64M-5 and 64M-2 antibodies recognized (6-4) photoproducts, but not normal DNAs, CPDs, or Dewar isomers [8]. The antigen-binding pockets of these antibodies were constructed so as to accommodate nucleotide dimers with T(6-4)T bases, in which the two base planes of the T(6-4)T segment were perpendicular to each other (Figure 2). T(6-4)T bases were capable of forming specific hydrogen bonds to the antibody Fab residues and placing their 3'-pyrimidone bases parallel to the Trp33H side-chain. The T(6-4)T segment was in a more compact conformation than standard B-DNA. It was in an extended conformation and adjacent bases are parallel to each other [37]. Therefore, standard B-DNAs could not be accommodated in the binding pocket of the Fab. When the CPD portion from the double-stranded DNA structure in complex with T4 endonuclease V [45] was superposed onto the location on the bound T(6-4)T segment, the folded bases crashed the Trp33H side-chain in a similar way. Therefore, CPD may not be accommodated in the antigen-binding site. Dewar isomers (Figure 1e) were produced from (6-4) photoproducts by irradiation with 313 nm ultraviolet [46], and the flap angle between the two mean planes of the four-membered rings in the bent 3' Dewar base was reported to be 117° in the solution structure of dT(Dewar)T of thymidylyl-(3'→5')-thymidine [47]. This bent 3' Dewar base may not have been sufficiently accommodated in the binding pocket nor placed parallel to the Trp33H side-chain of the Fab.

#### 3.1.2. Fab Interaction with the Double-Stranded DNA (6-4) Photoproduct

The binding affinities of the single-stranded DNA (6-4) photoproducts of various lengths were previously reported to be 4.5 × 10^6^~7.4 × 10^9^ M^−1^ for the 64M-5 Fab [9]. The higher affinity of the 64M-5 Fab was observed for longer oligonucleotides up to the hexamer, with each containing the T(6-4)T segment in the middle; however, the affinity of the octamer became virtually identical to that of the hexamer [9]. The binding affinity of the dAT(6-4)TA ligand was reported to be 3.1 × 10^8^ M^−1^, which was approximately 70 times higher than that for the dT(6-4)T ligand. In the structure of the 64M-5 Fab—ds-(6-4) DNA complex, the A8 base was sandwiched by the side chains of Tyr30L and Tyr100iH (Figure 4b and Figure 5). The N3 atom of the A8 base was hydrogen-bonded to the Tyr32L Oη atom. The His93L side chain interacted with the A11 base in a stacking manner. The O2P atom of the phosphate group connecting T10 and A11 was hydrogen-bonded to the Thr58H Oγ1 atom. These interactions between the antibody Fab and flanking nucleotides were responsible for the higher affinity of dAT(6-4)TA than dT(6-4)T.

The antibody 64M-5 was originally produced against single-stranded (6-4) DNA [8], and was later found to bind to double-stranded (6-4) DNA [13]. In the structure of the 64 M-5 Fab—ds-(6-4) DNA complex [14], 17 bp DNA with a one-base overhang was used as described. A loop comprised of several CDR L1 residues, including His27dL, Asn28L, and Tyr30L, was inserted between the flipped-out T(6-4)T-containing strand and complementary DNA strand (Figure 4). The loop insertion could occur concomitant with the flipping of DNA strands. The specific and extensive recognition of the photoproduct could be achieved by this separation. The surface area of DNA interfaced with Fab was 1052 Å^2^. In the case of the nucleotide-excision repair protein UvrB, the surface area of the protein to DNA was 1575 Å^2^ [48], which indicated the extensive interaction between the protein and DNA. In the case of CPD photolyase, the interface area of the protein to DNA was 1216 Å^2^ [49]. In the 64 M-5 Fab, the T(6-4)T segment and neighboring region were mainly recognized by the Fab. Most of this large area was attributed to the A8, T9(6-4)T10, and A11 nucleotides (674 Å^2^) and also to complementary T9, A10, and A11 (352 Å^2^) (Figure 4b). The A8 and T9(6-4)T10 nucleotides were in a flipped-out conformation due to the unraveling of base pairs. Nucleotide pairs other than these A8 and T9(6-4)T10 nucleotides were in the Watson-Crick base-pairing conformation. No hydrogen bonds were observed between the complementary DNA strand and CDR L1 residues. The nucleotides A10 and A11 in the complementary strand were located along the side of the CDR L1 loop. The interface area of these nucleotides and Fab was 216 Å^2^.

**Figure 6:**
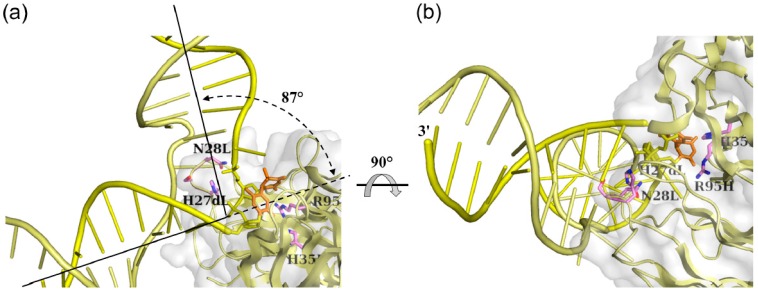

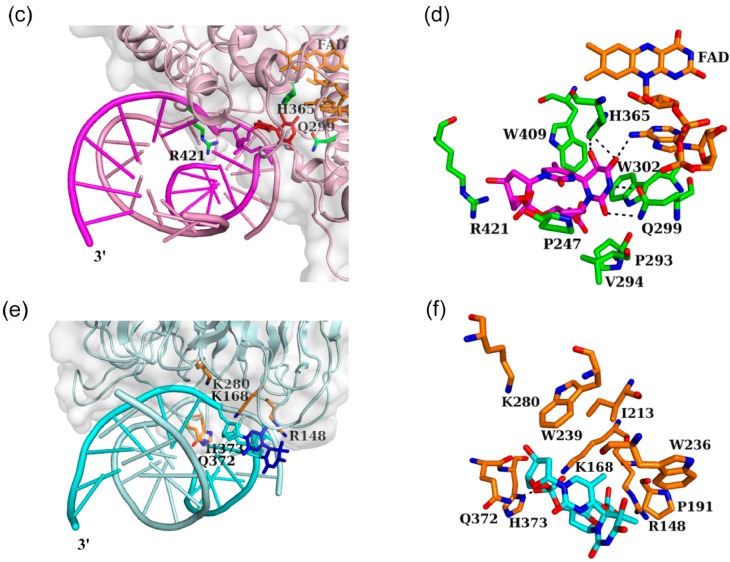
Structural differences in double-stranded (6-4) DNA in complex with the 64M-5 Fab, (6-4) photolyase, and the nucleotide excision repair protein DDB1-DDB2. DNAs are shown as cartoon models, and proteins are shown as cartoon and grey surface models. The T(6-4)T segments and some residues interacting with the T(6-4)T segments are shown as stick models. The complementary strand is shown in a paler color than the T(6-4)T-containing strand. The 3'-end of the T(6-4)T-containing strand is labeled as 3'. (**a**,**b**) Double-stranded (6-4) DNA in complex with the 64M-5 Fab (yellow). The base moiety of the T(6-4)T segment is colored orange. The view of (**b**) was almost the same as (**c**) and (**e**) if the T(6-4)T-containing strands were superposed between these structures; (**c**) Double-stranded (6-4) DNA in complex with the (6-4) photolyase (PDB ID: 3CVU, magenta). The base moiety of the T(6-4)T segment is colored red, and FAD is colored orange; (**d**) A close-up view of (**c**). Detailed interactions of the (6-4) photolyase (green) with the T(6-4)T nucleotide (magenta) are shown. Hydrogen bonds are shown in broken lines; (**e**) Double-standed (6-4) DNA in complex with the DDB1-DDB2 protein (PDB ID: 3EI1, cyan). The base moiety of the T(6-4)T segment is colored blue; and (**f**) A close-up view of (**e**). Detailed interactions of the DDB1-DDB2 protein (orange) with the T(6-4)T nucleotide (cyan) are shown. (Reproduced and slightly modified from reference [14] with the permission of the International Union of Crystallography.)

The nucleotides A8 and T9(6-4)T10 were flipped-out of the duplex, and were stabilized by interactions between the A8 base and side chains of Tyr30L and Tyr100iH and between the A11 base and His93L side chain (Figure 5). The CDR L1 loop including His27dL and Asn28L was inserted into the duplex as described above, resulting in a DNA kink angle of 87° (Figure 6a,b). The antibody 64M-5 was originally produced against single-stranded (6-4) DNA [8]. However, the 64M-5 Fab also bound double-stranded (6-4) DNA by partly separating the double strand. Thus, the antibody 64M-5 was considered suitable for the detection of both single- and double-stranded (6-4) DNA.

### 3.2. Interaction between DNA (6-4) Photolyase and the (6-4) Photoproduct

The (6-4) photoproduct is repaired by photoreactivation and nucleotide excision repair. Photoreactivation is performed using a (6-4) photolyase. It is proposed that the (6-4) photolyase repairs the (6-4) photoproduct by the catalytic proton transfer between a conserved histidine residue in the active site and the T(6-4)T segment, which is induced by the electron transfer from the excited flavin cofactor to the T(6-4)T segment [20,21,22,23,24]. In the crystal structure of double-stranded DNA containing the central T(6-4)T segment in complex with the DNA (6-4) photolyase [27], the (6-4) photolyase recognized the T(6-4)T segment completely flipped-out of the duplex (Figure 6c). Two nucleotides of T(6-4)T were flipped-out of the duplex, and all the remaining nucleotides retained Watson-Crick base pairing throughout the duplex. The 5'-thymine base of the T(6-4)T segment was hydrogen-bonded to the side chains of Gln299 and His365, while no hydrogen bonds were observed in the 3'-pyrimidone base (Figure 6d). The hydrophobic residues Pro247, Pro293, Val294, Trp302, and Trp409 formed the side wall of the pocket and interacted with the T(6-4)T bases. The Arg421 side chain protruded into the duplex, which resulted in setting apart the T(6-4)T segment. In the 64M-5 Fab, the side chains of Arg95H and His35H were hydrogen-bonded to the 5'-thymine and 3'-pyrimidone bases of the T(6-4)T segment, respectively (Figure 5 and Figure 6a,b). A major similarity between the 64M-5 Fab and (6-4) photolyase structures was that the 5'-thymine base was recognized by hydrogen-bonding and aromatic-aromatic interactions; however, the interacting residues are not conserved between the two proteins. Differences were observed in the orientation of the recognition of the T(6-4)T segment. The 64M-5 Fab recognized the entire segment of T(6-4)T containing bases and a sugar-phosphate backbone. In the (6-4) photolyase, the base moiety of the T(6-4)T segment was plunged into the binding pocket, and the sugar-phosphate moiety was left at the surface of the pocket (Figure 6c,d) because the (6-4) photolyase converts the T(6-4)T base into a normal TT base by transferring electrons from FAD located inside the protein (Figure 6c).

### 3.3. Interaction between Nucleotide Excision Repair Protein DDB1-DDB2 and the (6-4) Photoproduct

The nucleotide excision repair enzyme first excises and removes a short stretch of DNA containing the T(6-4)T base [26]. The nucleotide excision repair protein DDB1-DDB2 serves in the initial detection of the T(6-4)T lesion. In the crystal structure of double-stranded DNA containing the central T(6-4)T segment in complex with DDB1-DDB2 (Figure 6e), two nucleotides of T(6-4)T were flipped-out of the duplex, and all the remaining nucleotides retained Watson-Crick base pairing throughout the duplex [29]. The side chains of Arg148, Lys168, and Lys280 showed electrostatic interactions with the phosphate groups around the T(6-4)T segment, while no hydrogen bonds were observed in the T(6-4)T bases [29] (Figure 6e,f). The hydrophobic residues Pro191, Ile213, Trp236, and Trp239 formed a shallow pocket and were located near the T(6-4)T bases. The side chains of Gln372 and His373 forming a hairpin loop were inserted into the duplex, interacted with the complementary AA nucleotides as substitutes for the flipped-out T(6-4)T segment, and, thus, resulted in flipping of the T(6-4)T segment. A major similarity between the 64M-5 Fab and DDB1-DDB2 structures was that aromatic residues formed the T(6-4)T-binding pocket, and also that hairpin loops were inserted into the duplexes. However, the orientation of the T(6-4)T segment recognized by the DDB1-DDB2 protein was different from that recognized by the 64M-5 Fab. In DDB1-DDB2, the sugar-phosphate moiety of the T(6-4)T segment was oriented toward the surface of the binding pocket, while the base moiety was oriented toward the outside (Figure 6e,f). This orientation was plausible for the nucleotide excision repair proteins which would not necessarily need to recognize the base moiety of the T(6-4)T segment. That contrasts to (6-4) photolyases which recognize the base moiety.

### 3.4. Distinctive Binding-Site Structures of Proteins Interacting with (6-4) Photoproducts

Structural comparisons of these protein structures in complex with double-stranded (6-4) DNA revealed that the antibody 64M-5, photoreactivation enzyme (6-4) photolyase, and nucleotide excision repair protein DDB1-DDB2 had distinctive binding-site structures that were appropriate for their functions. In all of these complex structures, the T(6-4)T segment was flipped-out of the duplex and the DNA helices were kinked at the T(6-4)T segment. Since DNA bending angles of 44° and 27° were reported in the solution structures of uncomplexed double-stranded DNA containing the T(6-4)T segment [39,40,41], the covalent linkage of T(6-4)T was attributed to the kink in double-stranded DNA. The DNA bending angles of 48° for (6-4) photolyase and 34° for DDB1-DDB2 were similar to those of the solution structures of the DNA duplex-decamer containing T(6-4)T. The sharp kink of 87° observed for the 64M-5 Fab was attributed to the intensive interaction between the complementary strand and CDR residues. This antibody was originally established for the detection and quantification of the (6-4) photoproduct [8]. However, the antibody may be substituted for other repair proteins in order to decrease the mutagenic activity of the (6-4) photoproduct by binding tightly to the (6-4) photolesion.

Although the T(6-4)T segments in the solution structures were not flipped-out, but partly hydrogen-bonded to the complementary strand, the flip-out was commonly observed in the crystal structures of 64M-5 Fab, (6-4) photolyase, and DDB1-DDB2. Therefore, the flipped-out structure represented one of the possible conformations of double-stranded (6-4) DNAs. Double-stranded (6-4) DNA showed diminished intrastrand stacking and interstrand hydrogen bonding interactions around the T(6-4)T segment, and allowed for the insertion of the hairpin loop and flipping out of the T(6-4)T segment. In the absence of the T(6-4)T segment, however, intact base stacking and hydrogen bonding interactions would show an energy barrier that prevents the insertion of the hairpin loop [29]. The T(6-4)T segment destabilizes the double-stranded DNA structure, and facilitates the flip-out of the T(6-4)T segment. In the structure of CPD photolyase-duplex DNA, the dinucleotide containing the CPD lesion was flipped out of the duplex [49]. This flipped-out conformation rather than the base-paired conformation was appropriate for recognition by proteins. When the T(6-4)T lesion is formed, the base-paired conformation is almost broken, and thus the lesion may not have a chance to form Watson-Crick type hydrogen bonds with the complementary bases. Based on structural similarities between double-stranded (6-4) DNAs in complex with proteins, the flip-out and DNA kink were found to be common features of (6-4) photoproducts recognized by proteins.

## 4. Conclusions

(6-4) Photoproducts are the major constituents of ultraviolet-damaged DNAs, and are more mutagenic than CPDs [3]. In the crystal structures of the 64 M-2 Fab—dT(6-4)T [11], 64 M-2 Fab—dTT(6-4)TT [12], and 64 M-5 Fab—ds(6-4) DNA complexes [14], dT(6-4)T adopted a closed circular structure by forming a covalent bond between the C6 atom of the 5'-base and C4 atom of the 3'-base. The 5'-side thymine base of the T(6-4)T segment was in a half-chair conformation while the 3'-side pyrimidone base was in a planar conformation. Both base planes were nearly perpendicular to each other. In the solution structure of the DNA duplex-decamer, which contained dT(6-4)T/dAA nucleotide pairs, all nucleotides, except for the 3'-side pyrimidone of the dT(6-4)T segment, retained hydrogen bonds with complementary bases, and the DNA duplex flanking the T(6-4)T segment was kinked by 44°. The covalent link in the (6-4) photolesion accounted for the kink in double-stranded DNA. In the structure of the 64M-5 Fab—ds(6-4) DNA complex [14], the side chains of Arg95H and His35H were hydrogen-bonded to the T(6-4)T bases. The aromatic side chain of Trp33H was in a stacking interaction with the 3'-pyrimidone bases of the T(6-4)T segment. These interactions were appropriate for the compact and unique structure of the T(6-4)T segment described above. A common feature in the three structures of 64M-5 Fab, (6-4) photolyase, and nucleotide excision repair protein DDB1-DDB2, was that aromatic residues interacted with the T(6-4)T bases. However, major differences were observed in the interacting orientation of the recognition of the T(6-4)T segment. These proteins had distinctive binding-site structures that were appropriate for their functions. In the structure of the 64M-5 Fab, three nucleotides of T(6-4)T and 5'-side adenosine were flipped-out of the duplex, and the duplex was sharply kinked by 87°. The insertion of the CDR L1 loop between the T(6-4)T segment and complementary strand accounted for the steep kink and resultant strand separation. In the structures of (6-4) photolyase and DDB1-DDB2, two nucleotides of T(6-4)T were flipped-out of the duplex by the insertion of a hairpin loop. These flipped-out conformations were common between these structures and appropriate for recognition by these proteins.
